# The plight of COVID-19 pandemic on medical students and residency applicants

**DOI:** 10.1016/j.amsu.2020.10.010

**Published:** 2020-10-10

**Authors:** Ezza Fatima Tariq, Prem Kumar Sah, Adnan Malik

**Affiliations:** Multan Medical & Dental College, Southern Bypass Road, Multan, Pakistan; Department of Health Services, Ministry of Health and Population, Government of Nepal, Ramshahpath, Kathmandu, Nepal; Floyd Medical Center, Rome, GA, USA

**Keywords:** Pandemic, COVID-19, Medical students, Education, Tele-health, Residency

## Abstract

In this pandemic situation, medical students find them in a state of apprehension. With medical institutions closed and switch to online teaching and telehealth, many aspects of medical learning are still compromised, including core clinical rotations, clinical skills evaluation, and exams cancelation. The medical students are distressed about their continuity of education and developing the necessary skill to feel confident enough to practice in the future. The involvement of medical students as frontline workers with inadequate clinical training, the uncertainty of future, lack of knowledge, and access to personal protective equipment have aroused a sense of fear in them.

They not only nurture their clinical skills from the clinical rotations but it also helps adapt to their residency program later. With the lack of clinical experience, challenges of online learning, cancelation of conferences, and on-site research, medical students are struggling to make their residency application competitive. Recruiting residents amid the pandemic is a difficult task. The uncertainty in the unprecedented situation has an immense psychological impact on medical students and residency applicants. Despite the hurdles being faced, there are many ways where medical students and residency applicants can use their knowledge to help in fighting the pandemic. They can volunteer in the field of research on COVID-19, as a contact tracer, and provide peer support to the patients through telecommunications. Many avenues are being sought to ensure the continuation of medical education. However, how efficient these methods will prove in the future is yet to be revealed.

## Highlights

• Adverse effects of COVID-19 on medical students and residency applicants.
• Online methods for teaching is promising yet with certain reservations. Improvised online learning, creation of a new communication channel to educate will allow them to work effectively.
• Fear of the infection, financial burden, and academic uncertainties causes stress in medical students.
• Medical students can volunteer in capacities affording minimum patient contact.
• Regulation authorities trying to find ways to substitute in person interviews and in-patient hospital rotations for residency applicants. Challenges are faced by residency applicants despite the solutions found through virtual platform.

## 1 Introduction

Living through a pandemic situation comes with all sorts of burdens in addition to health care. It adds to psychological and financial difficulties. One particular group significantly affected includes medical students and residency applicants. Especially the students in pre-clinical and clinical years of medical education are adversely affected. The need of the times is to prioritize safety and minimum exposure of students to the COVID 19 positive population. The steps taken include cancelation of core clinical rotation, problem-based learning, in-person anatomy dissections, group learning session, and conferences. These methods have been an essential part of grooming the students into the physicians they become in the future. However as safety becomes the need of the hour, these aspects have to be compromised upon raises concern about their safety, education, and their roles in taking care of the patient.

## 2 Online education for medical students; adaptability is the need of the time

It is a blessing that we live in a technologically advanced era. It has allowed us to find effective alternatives to continue medical education via the online platform. Students can continue to take lectures from the safety and comfort of their homes. Some developed countries have started offering online elective rotations which is a creative way of bridging the gap of clinical exposure offered to students as in-hospital rotations. These adaptations to the present situation sound promising. However, in the present situation, there is a concern that how efficiently virtual clinical exposure can replace the hands-on experience for the students? Will these students feel confident enough to practice medicine as they graduate [1]?

Adaptability is by all means the necessity skill at the moment, the student who will adapt to new changes and evolving methodologies will have an advantage over who fails to do so. Shift to an online platform is a very attractive avenue. This, at the same time, causes a disparity for students belonging to low socio-economic status especially in third-world countries. For example, in countries like Pakistan and Nepal, the students who belong to rural areas do not have internet access and are struggling to keep up with fellow students who can afford the utility. For students with such backgrounds living in hostels and studying in the library and using on-campus internet and resources has been a very important aspect of their educations. Delay in graduation is a concern for such students as paid internship affords them the financial security for the future [2]. This observation is supported by a study by Cao et al. [3] which reported that students who have financial stability and come from urban backgrounds have less anxiety than their fellow medical students.

Similarly, postponing examinations for an indefinite period has raised anxiety and apprehension in the students. Many final year students have their graduation on hold. It may be a cause of financial concerns, as many would like to be in a financially sound position as they start their paid internships [2].

Moreover, as teaching has moved online so have the testing methods. The final year examination and post-graduation examination have been postponed. These exams have been important in distinguishing the students on merit. Some countries have proposed online testing, in the form of open-book exams, as a solution to the continuation of the education curriculum. This raises concerns about the moral responsibility of the students and the efficiency of the method in identifying high merit students. Despite the reservations, this method is the sole reasonable middle way for tackling the present situation [4].

Another issue with online learning faced by students is that learning from home has led to difficulty in creating a healthy work-life balance In the pre-pandemic days, students had the chance to create a healthy work-life balance by dividing their time and focus on education and healthy family and friends oriented activities [5]. Now working from home has blurred this distinguishment and has increased anxiety.

The uncertainty of the future, which is already competitive, and the fear of losing dear ones, is a real concern in the students. This has led to psychological impacts that cannot be ignored. This was demonstrated in a study by Meo et al. [6] which investigated the impact of quarantine on the mental wellbeing and learning behaviors of medical students in King Saud University, Riyadh, Saudi Arabia. Out of the sample size of 530 students including male and female, 44.1% showed emotional detachment from the family and friends, 23.5% felt depressed, 38.1% reported feeling emotionally drained and 56.2% reported a decrease in the overall study time. The study concluded that the students showed emotional detachment from their family and friends with a decrease in the overall study time and performance. This study consolidates the fact that studying is difficult amidst the horror of the pandemic. This fact should be kept in mind while designing educational activities as we would not want to put the mental health of the medical students at risk.

## 3 Is recruiting medical students on frontline to fight pandemic worth it?

In light of the need for the increased demand of healthcare providers in the hospitals, many medical schools suggested increasing the workforce by deploying final-year medical students on the front line. Some medical schools suggested early graduation of final year students to meet the demand. No matter how attractive this suggestion sounds, a lack of enough clinical experience would threaten patient care. Moreover, deficient personal protective equipment poses a continuous threat of exposure to their family members, leading to increased anxiety and depression [7,8]. These steps further add to the worry of the students even though a large number of students would want to volunteer and contribute.

Carrascossa et al. [9] conducted a cross-sectional study on medical student internships in different medical colleges of Brazil. The sample included 317 students of the intern year irrespective of the gender, from the public and private institutions. The study aimed to assess the apprehensions of these students regarding the change of study pattern, the stress of the COVID-19 exposure, and the uncertainty of the future. According to the study, 50.2% reported interruption of their study, 71.6% reported difficulty in access to personal protective equipment, 44.2% did not feel safe while performing patient care, 80.8% confessed fear of contracting COVID 19 and 50% felt insecure about their future. The study demonstrated that there is a fear in students approaching COVID 19 positive patients and have concerns about having adequate personal protective equipment.

## 4 How can medical students contribute to fighting the pandemic?

In such a situation the best solution is to keep the off-site educational activities intact and find avenues for medical students to volunteer in capacities where minimum exposure to sick is guaranteed. .Using the knowledge the medical students have can be an asset in areas where there is no contact with the patients. They can volunteer, use social media to educate the masses about social distancing and the SOP (Standard Operating Procedure) that need to be followed. They can help as contact tracers and also provide peer support for patients in isolation. They can be valuable in fields of research on COVID-19, as there is still so much we do not know about the pathogen and the disease [10]. Being a productive part of a society and helping within their capacity will positively impact their mental health. They will have the opportunity to understand the emotional, psychological, and social aspects of patient care.

## 5 The Dilemma for residency applicants; virtual interviewing as a proposed solution

Many students who planned to apply for a residency program this year found themselves in a much higher dilemma. With clinical rotations and exams canceled; the students do not know how to approach these situations. In some parts of the world like Pakistan, the bi-annual induction is postponed, leading to a challenging residency induction. In Nepal, the institution of the standard residency entrance examination, postponement of the academic year, and the cancelation of the licensing exam have created uncertainty in the students' career.

American Medical Association suspended United States medical licensing exams indefinitely to abide by social distancing rules and maintain the flattening of the curve. Among this is the USMLE step 2 clinical skill exam, which allows the assessment of clinical skills, English proficiency, and interpersonal skills. This led to a lot of concern among the students. However, a multiple pathway suggestion has been put forward, but we do not know its efficacy [11].

Clinical rotations have helped students develop clinical insight esp. for the international medical graduates which provide them the opportunity to get accustomed to the US Healthcare system. This also gives them a chance to earn a strong letter of recommendation or even have a chance of matching at the same institution. It also fulfills an important aspect of their residency application. Unavailability of the essential clinical rotations for a competitive match application would be a disadvantage for these medical students compared to those who have completed their rotations before the pandemic. There is a significantly high chance of students matching at the same institute, they rotated; 57% of the students from the home institution and 44% of the students on away rotation matched in institutions they rotated [[11], [12], [13], [14]].

AAMC and ACGME suggested on May 14, 2020, to conduct virtual interviews for residency candidate recruitment [14]. This step assures the safety and maintenance of flattening of the curve for COVID-19. Many students, natives, and international medical graduates alike would have to borrow money to afford the travels across the country to interview previously; they will have the chance to avoid that expenditure. It is still at the expense of many opportunities the candidates had in case of in-person interviews.

Kenigsberg et al. [14] conducted a survey among 2019 and 2020 Urology residency matches about their attitude towards the shift to virtual interviews. Out of 156 applicants who responded, 81% reported that the faculty interview can be virtual and 64% reported that interaction with the residents is an important part of the interview day. Moreover, 46% reported pre/post interview social and 51% believed city visits have a large impact on their rank list. The study concluded that the faculty interview can be conducted virtually while the resident's interaction cannot be replicated virtually; interaction and social provide an opportunity to the program and the residency applicants get to know each other better where they will be spending their 3–7 years of training.

The concerns still arise about how effective this will be in comparison to conventional in-person interviews. In-person interviews allowed applicants to make educated and well inform choices about their rank order and for the institution to endorse their strengths and teaching methods at the same time. Like the many adoptions made to the current situation, this allows continuity of the process of residency candidates recruitment but with the aforementioned reservations [15].

## 6 What residency applicants can do at this time?

It is a high time residency applicants demonstrate adaptability to the ever-changing situation in light of the pandemic. The aspirants can improve their CV by volunteering, demonstrating empathy, and readiness to help when it is demanded of them. Participating in remote research, review articles, and systemic review and meta-analysis are good options while on-site laboratories and related work have come to a halt.

The new mode of interviewing will be new to many. It is worthwhile to get accustomed to a new web conferencing application and practice virtual interviewing [11].

## 7 Conclusion

Medical students and residency applicants are being affected in various ways due to the present pandemic. The steps should be taken t o involve them in providing support with minimum exposure to sick patients. Persistence and responding positively to the continuous change in this time will prove fruitful for the students. While a lot of support and judicious policies are expected from the authorities to facilitate them; many innovative ideas are being explored to avoid hampering continuity of education and their medical career but with unproven efficiency.

## Declaration of competing interest

There is no conflict of interest.

## Ethical approval

Not applicable.

## Funding sources

The study has not received any funding.

## Provenance and peer review

Not commission, externally peer reviewed.

## Consent

Not applicable.

## Author contribution

Ezza Fatima Tariq was involved in the study concept, design, literature search, manuscript preparation, data collection, data analysis, and manuscript writing.

Prem Kumar Sah was involved in manuscript writing, manuscript edit, literature review, data analysis, revision with relevant reference and preparation of the manuscript for submission.

Adnan Malik provided support and mentorship for its development, and revision of the manuscript.

## Registration of Research Studies

Name of the registry: Not applicable

Unique Identifying number or registration ID: Not applicable

Hyperlink to your specific registration (must be publicly accessible and will be checked): Not applicable

## Guarantor

Prem Kumar Sah. He is the second author and the corresponding author for this manuscript.

## Annals of medicine and surgery

The following information is required for submission. Please note that failure to respond to these questions/statements will mean your submission will be returned. If you have nothing to declare in any of these categories then this should be stated.

## Please state any conflicts of interest

There is no conflict of interest.

## Please state any sources of funding for your research

The study has not received any funding.

## Ethical approval

Not applicable.

## Consent

Not applicable.

## Author contribution

Ezza Fatima Tariq was involved in the study concept, design, literature search, manuscript preparation, data collection, data analysis, and manuscript writing.

Prem Kumar Sah was involved in manuscript writing, manuscript edit, literature review, data analysis, revision with relevant reference and preparation of the manuscript for submission.

Adnan Malik provided support and mentorship for its development, and revision of the manuscript.

## Registration of research studies

1. Name of the registry: Not applicable.

2. Unique Identifying number or registration ID: Not applicable.

3. Hyperlink to your specific registration (must be publicly accessible and will be checked): Not applicable.

## Guarantor

Prem Kumar Sah. He is the second author and the corresponding author for this manuscript.
